# DAT: Deep Learning-Based Acceleration-Aware Trajectory Forecasting

**DOI:** 10.3390/jimaging10120321

**Published:** 2024-12-13

**Authors:** Ali Asghar Sharifi, Ali Zoljodi, Masoud Daneshtalab

**Affiliations:** 1 School of Innovation, Design and Technology (IDT), Mälardalen University, 72123 Västerås, Sweden; sharifinjf@gmail.com (A.A.S.); ali.zoljodi@mdu.se (A.Z.); 2 Department of Computer Systems, Tallinn University of Technology, 19086 Tallinn, Estonia

**Keywords:** end-to-end trajectory forecasting, deep learning, perception, acceleration prediction

## Abstract

As the demand for autonomous driving (AD) systems has increased, the enhancement of their safety has become critically important. A fundamental capability of AD systems is object detection and trajectory forecasting of vehicles and pedestrians around the ego-vehicle, which is essential for preventing potential collisions. This study introduces the Deep learning-based Acceleration-aware Trajectory forecasting (DAT) model, a deep learning-based approach for object detection and trajectory forecasting, utilizing raw sensor measurements. DAT is an end-to-end model that processes sequential sensor data to detect objects and forecasts their future trajectories at each time step. The core innovation of DAT lies in its novel forecasting module, which leverages acceleration data to enhance trajectory forecasting, leading to the consideration of a variety of agent motion models. We propose a robust and innovative method for estimating ground-truth acceleration for objects, along with an object detector that predicts acceleration attributes for each detected object and a novel method for trajectory forecasting. DAT is trained and evaluated on the NuScenes dataset, demonstrating its empirical effectiveness through extensive experiments. The results indicate that DAT significantly surpasses state-of-the-art methods, particularly in enhancing forecasting accuracy for objects exhibiting both linear and nonlinear motion patterns, achieving up to a 2× improvement. This advancement highlights the critical role of incorporating acceleration data into predictive models, representing a substantial step forward in the development of safer autonomous driving systems.

## 1. Introduction

Accurately forecasting the future movements of objects surrounding autonomous vehicles (AVs) is vital for safe driving and collision avoidance. As driving involves numerous unpredictable factors, such as other vehicles and pedestrians, AV systems must anticipate their potential trajectories to ensure safe operation [1,2,3].

In this paper, we aim to address the inherent limitations present in previous end-to-end trajectory forecasting methodologies, especially in forecasting agents with nonlinear motions [4,5]. Existing approaches are effective in estimating the behavior of static agents and, to a certain degree, agents that follow a linear motion model. However, these methods are inadequate when it comes to dealing with targets characterized by nonlinear dynamics because they often depend on a constant speed equation that fails to accurately capture the nuanced behavior of such objects.

To address this, our proposed model places a specific emphasis on learning parameters essential for modeling targets with both constant acceleration and constant speed. This enhancement not only proves effective for agents with nonlinear motions but also significantly improves forecasting accuracy for agents with linear motions. It is noteworthy that the incorporation of object acceleration and velocity goes beyond trajectory forecasting, extending its utility to applications such as decision-making and collision avoidance.

The absence of acceleration data for objects is a common limitation in existing datasets [6,7,8]. In response to this challenge, we extend our dataset by adding an acceleration feature computed using a second-order regression (SOR) [9] method and then train our model to predict acceleration for all target objects at each time point. Our key contributions can be succinctly summarized as follows:

- Novel end-to-end solution for advanced motion model acquisition (main contribution): Our model is designed to learn and estimate the acceleration and velocity of every detected object, providing a comprehensive understanding of the dynamic nature of the targets.
- Supervised acceleration prediction: To calculate acceleration, we employ an SOR method. This choice is motivated by the presence of inherent noise in our sensor data and labeled ground truth, as highlighted by Xu et al. [10], Gu et al. [11]. This ensures the robustness, reliability, and accuracy of our model’s forecasting.

## 2. Related Work

**Figure 1:**
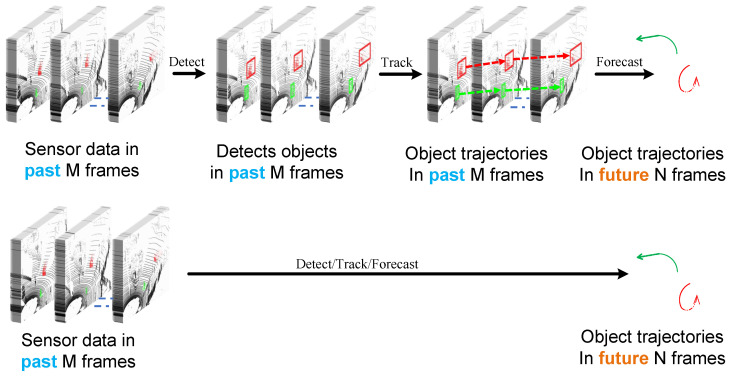
(**Top** Row) Cascade methods, which handle detection, tracking, and forecasting in a sequential pipeline, are vulnerable to error propagation. In the diagram, the arrows indicate the direction of processing for the Lidar data, moving from raw input to the final output. The input data, represented in blue, is gathered from past observations, while the future output is shown in orange. This is because each stage assumes error-free input from the previous one, which is often unrealistic in real-world applications. As a result, errors can accumulate and negatively impact the final predictions. (**Bottom** Row) End-to-end methods, on the other hand, directly predict future trajectories from raw data. This unified approach allows for the joint optimization of detection, tracking, and forecasting, leading to more accurate and reliable results.

This section offers a concise review of trajectory forecasting techniques that rely on point cloud data. We commence by exploring traditional methods, which involve a cascaded approach of object detection, tracking, and trajectory prediction. This sequential process is illustrated in the top row of Figure 1. Subsequently, we delve into cutting-edge approaches that unify these tasks into a single, end-to-end framework, as depicted in the bottom row of Figure 1.

### 2.1. Cascade Approaches

Conventional self-driving systems address the autonomy challenge by breaking it down into three primary tasks: object detection, object tracking, and motion prediction. These tasks are typically handled by separate modules operating in a sequential manner, with each component being trained independently and uncertainties propagated through the system [2]. Such approaches assume that the precise trajectories of agents are known. By analyzing trajectory data over a limited timeframe, predictions about future movements can be generated. For example, datasets such as NuScenes [8] and Argoverse [7] provide annotated trajectory data to support this forecasting process.

Numerous methods discussed in the literature utilize neural networks, particularly, recurrent neural networks (RNNs), which are designed to explicitly incorporate the historical states of agents into their analysis [12,13]. RNNs and their extensions, including Long Short-Term Memory (LSTM) networks and Gated Recurrent Units (GRUs), utilize a single hidden state vector to store all temporal information. This unified memory representation makes it possible to access the memory as a whole but limits the ability to retrieve specific elements of stored knowledge [3]. The Memory-Augmented Neural Trajectory predictor (MANTRA) [3] introduces an external associative memory mechanism designed to retain essential and non-redundant trajectory data. Unlike models relying on a single hidden state for memory representation, MANTRA enables element-wise addressing, allowing the selective retrieval of specific, relevant information during execution.

Forecasting relies on both spatial and temporal features, as these two components collectively offer a comprehensive perspective on the potential actions of target objects and the likelihood of those actions occurring. Studies such as [14,15,16] employ rasterization techniques to represent both agents and high-definition map features, converting elements like lanes and crosswalks into colored lines and polygons. However, this rasterized approach results in a highly intricate depiction of the environment and agent history, demanding substantial computational resources and extensive data for both training and deployment. To mitigate this issue, VectorNet [17] introduces a vector-based representation designed to capture the spatial relationships of individual road elements using graph neural networks. LaneConv [18] develops a lane graph using vectorized map data and introduces LaneGCN to model the topological structure and long-range dependencies between agents and map information. VectorNet [17] and LaneConv [18] can be seen as adaptations of graph neural networks for prediction, demonstrating a robust ability to capture spatial locality. To integrate both spatial and temporal learning within a cohesive and adaptable framework, Ye et al. [19] introduced Temporal Point Cloud Networks (TPCN). TPCN frames the forecasting task as a combined learning process involving both a spatial module and a temporal module.

Transformer-based models demonstrate performance that is either comparable to or surpasses that of other network architectures, such as convolutional and recurrent neural networks, across various visual benchmark tasks. Due to its notable effectiveness and reduced requirement for vision-specific inductive bias, transformers are garnering increased attention within the computer vision community [20]. Yuan et al. [21] propose a new transformer-based trajectory forecasting model that trains the time and social dimensions. The proposed method facilitates a direct influence of one agent’s state at a specific time point on the state of another agent at a subsequent time, thereby modeling the dynamic interdependencies between agents over time. In a separate study, Khandelwal et al. [22] propose a Recurrent Neural Network (RNN) approach designed for context-sensitive multi-modal behavior prediction. The input to this model incorporates a road network attention mechanism alongside a dynamic interaction graph, enabling it to capture both meaningful geometric and social connections.

Cascade approaches to trajectory forecasting, as mentioned, are developed in isolation from their upstream perception modules (detection and tracking). They operate under the assumption of accurate past trajectory information. Therefore, their performance significantly diminishes when using real-world tracking results, which are often noisy. This is primarily caused by the propagation of errors, including noisy tracks, fragmented trajectories, and identity switches, from the tracking to the forecasting stage [23]. A novel forecasting framework introduced by Weng et al. [23] employs affinity matrices as input, rather than tracklets. This innovation reduces the likelihood of errors stemming from data association and facilitates the transfer of more detailed information to the forecasting process.

### 2.2. End-to-End Approaches

To mitigate the spread of errors and enhance inference efficiency in conventional approaches, where learning occurs independently, researchers such as Wang et al. [24,25], Guo et al. [26], Yin et al. [27], Li and Guivant [28], Simon et al. [29] have explored the integration of detection and tracking in an end-to-end framework. In a similar vein, Weng et al. [30] introduced a network that parallelized tracking and forecasting tasks through the use of a Graph Neural Network.

To the best of our knowledge, Fast and Furious (FaF) [31] represents the pioneering deep neural network capable of concurrently executing 3D object detection, tracking, and motion prediction using data derived from 3D sensors. However, the forecasting horizon of [31] was restricted to a mere 1 s. In comparison, IntentNe [32] extends the prediction scope to estimate future high-level driver behaviors. Ref. [33] further advances this approach by integrating detection, forecasting, and motion planning into a single framework. In addition, Zeng et al. [33] present a novel perception loss that promotes the generation of precise 3D detections and motion forecasts by the intermediate representations. All aforementioned methods disregard the statistical dependencies between agents, opting instead to independently predict each trajectory based on given features. Li et al. [1] introduced a novel network architecture that explicitly modeled the interactions among agents.

Weng et al. [34] propose an alternative approach to the detect-then-forecast pipeline by reversing its sequence. Instead of adhering to the traditional process of first detecting and tracking objects before forecasting, their method begins with predicting future states. Object detection and tracking are subsequently applied to the projected point cloud sequences to determine future positions. An important strength of that method is its ability to deliver a detailed representation of forecasting by integrating information about both foreground and background objects within the scene. In a similar vein, FutureDet [4] focuses on directly predicting future object locations rather than modeling the evolution of point cloud sequences over time. This approach incorporates backcasting to determine the initial position of each trajectory. By aligning backcasted future predictions with current detections in a many-to-one way, FutureDet effectively captures a distribution of multiple potential future states. In a different approach, TrajectoryNAS [35] utilizes Neural Architecture Search (NAS) to systematically optimize the architecture, significantly improving both accuracy and efficiency compared to traditional approaches. That method achieves higher accuracy and lower latency.

End-to-end approaches often assume a constant velocity over the time elapsed between frames in a LiDAR sequence. This assumption increases the error in predicting the next location of target objects, especially for those with nonlinear motions. In this work, we propose DAT, an end-to-end trajectory forecasting model that predicts acceleration values, thereby reducing potential errors in predicting the objects’ next position and providing more accurate forecasting of their trajectories.

## 3. Methodology

Various factors need to be addressed to forecast the trajectories of vehicles and pedestrians surrounding the autonomous vehicle. One of the key factors is the speed and acceleration of surrounding objects. State-of-the-art trajectory forecasting models [1,4,31,33] assume that objects do not experience acceleration between frames and utilize the constant speed motion model for different tasks. However, vehicles adjust their speeds dynamically based on the environment and interactions with other agents, employing acceleration or deceleration mechanisms. Consequently, real-world situations often differ from the conventional assumption of constant speed, with acceleration being a predominant factor. These assumptions can lead to significant inaccuracies in the predicted locations of objects. To address this issue, we continuously predict objects’ acceleration between captured frames and employ a constant acceleration model, resulting in much more accurate predictions of object locations.

In the available datasets for self-driving cars, such as NuScenes [8] and ArgoVerse [7], the acceleration feature is not present as the ground truth. To supervise the training of DAT to predict the acceleration of target objects, which can be used in trajectory forecasting, we need to extend these datasets with the acceleration ground truth. We compute the acceleration of each target object using the SOR elaborated in Section 3.1.

(1) $$y=\frac{1}{2}at^{2}+v_{0}t+y_{0}$$

(2) $$a=\frac{2(y_{2}-y_{0})}{\left(t_{2}-t_{1}\right)\left(t_{2}-t_{0}\right)}-\frac{2(y_{1}-y_{0})}{\left(t_{1}-t_{0}\right)\left(t_{2}-t_{1}\right)}$$

(3) $$v_{0}=\frac{2\left(y_{1}-y_{0}\right)\left(t_{2}-t_{0}\right)}{\left(t_{2}-t_{1}\right)\left(t_{1}-t_{0}\right)}-\frac{2\left(y_{2}-y_{0}\right)\left(t_{1}-t_{0}\right)}{\left(t_{2}-t_{1}\right)\left(t_{2}-t_{0}\right)}$$

(4) $$\sigma_{a}^{2}=\frac{8\sigma_{y}^{2}}{{\left(t_{2}-t_{1}\right)}^{2}{\left(t_{2}-t_{0}\right)}^{2}}+\frac{8\sigma_{y}^{2}}{{\left(t_{1}-t_{0}\right)}^{2}{\left(t_{2}-t_{1}\right)}^{2}}$$

(5) $$\sigma_{V_{0}}^{2}=\frac{8\sigma_{y}^{2}{\left(t_{2}-t_{0}\right)}^{2}}{{\left(t_{2}-t_{1}\right)}^{2}{\left(t_{1}-t_{0}\right)}^{2}}+\frac{8\sigma_{y}^{2}{\left(t_{1}-t_{0}\right)}^{2}}{{\left(t_{2}-t_{1}\right)}^{2}{\left(t_{2}-t_{0}\right)}^{2}}$$

### 3.1. Deriving Acceleration Features

Acceleration is calculated by analyzing the coordinates of points. This process is employed to estimate acceleration under the assumption that objects maintain constant acceleration during short time intervals. Equation (1) represents the mathematical formulation of the constant acceleration motion model. By utilizing the positions of an agent at three designated points $\left(t_{1},y_{1}\right),\left(t_{2},y_{2}\right)$, and $\left(t_{3},y_{3}\right)$, we can extract the values of acceleration and velocity according to Equation (2) and Equation (3), respectively. These derived values effectively model the motion behavior of agents experiencing constant acceleration.

Using Equations (2) and (3) to calculate *a* and $v_{0}$ yields accurate results if the position and time values are highly precise and free from noise. However, due to the short time intervals between samples (0.1 s, 0.2 s), even minor errors in measuring positions can lead to significant variations in the calculated acceleration and initial velocity. Equations (4) and (5) express the variances $\sigma_{a}^{2}$ and $\sigma_{V_{0}}^{2}$ in terms of the position error variance $\sigma_{y}^{2}$ (see Appendix A), illustrating that small errors in position measurements result in substantial inaccuracies in acceleration and initial velocity. In practice, factors such as sensor intrinsic noise and labeling errors contribute to measurement inaccuracies [10,11]. Consequently, an object’s calculated speed and acceleration can vary significantly between different frames, contradicting the laws of physics and the inherent nature of physical bodies.

For instance, in the conducted simulation, a car was modeled with constant acceleration motion and initial motion parameters set as acceleration $a=1.5\;{m/s}^{2}$, initial velocity $v_{0}=5\;m/s$, and initial position $x_{0}=10\;m$, representing a typical CAN Bus scenario [8]. The car was assumed to be accelerating, and Gaussian noise with a mean of zero and a variance of $0.01\;m^{2}$ was added to the position data. The NuScenes dataset specifies the LiDAR range accuracy as 2 cm, and the accuracy of the Inertial Navigation System (INS) and Global Positioning System (GPS) positions is 20 mm [8]. It was further assumed that 10 position samples were recorded per second, with a window length of 10 samples used for acceleration calculation.

**Figure 2:**
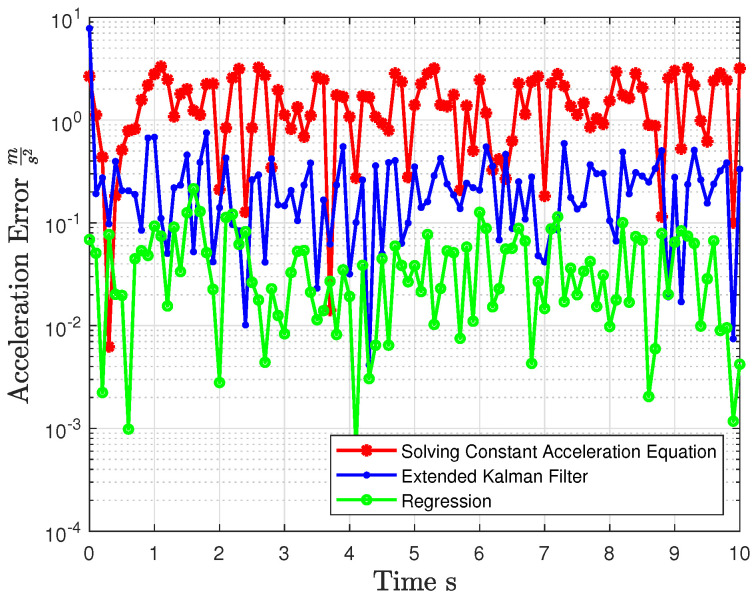
Acceleration error comparison across different methods.

**Figure 3:**
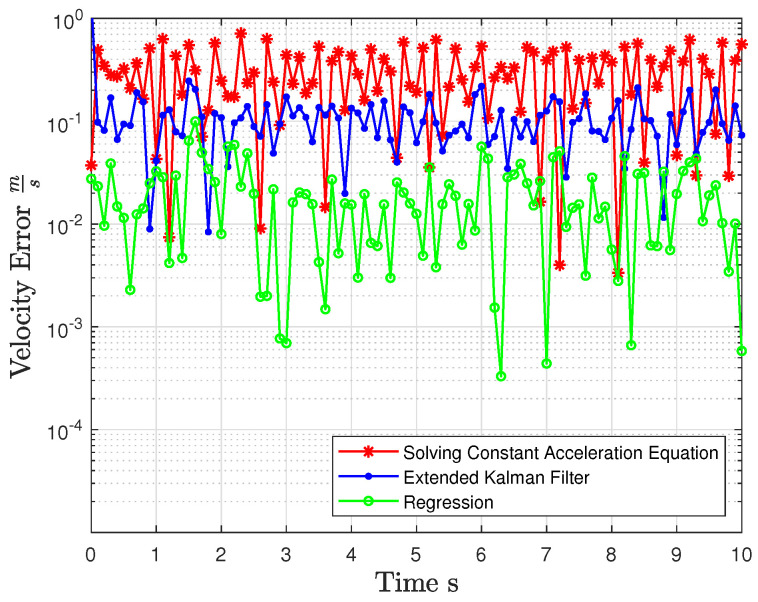
Initial velocity error comparison across different methods.

Speed and acceleration were computed using the specified equations, and their errors relative to the ground truth are depicted in Figure 2 and Figure 3. The mean and variance of these errors compared to the ground truth are presented in Table 1, highlighting the deviation from the actual values.

To address this issue, we utilized two fundamental baselines: the Extended Kalman Filter (EKF) [36] and SOR [9], both of which are robust against noise.

#### 3.1.1. EKF

Acceleration estimation can be achieved using the Extended Kalman Filter (EKF) [36]. The state vector, encompassing position, velocity, and acceleration, is defined in Equation (6). The state transition matrix F and control input matrix G are specified in subsequent equations.

(6) $$x={\left[\begin{array}{ccc} y & v & a \end{array}\right]}^{T}$$

(7) $$F=\left[\begin{array}{ccc} 1 & \Delta t & \frac{\Delta t^{2}}{2}\\ 0 & 1 & \Delta t\\ 0 & 0 & 1 \end{array}\right]$$

(8) $$G={\left[\begin{array}{ccc} \frac{\Delta t^{2}}{2} & \Delta t & 1 \end{array}\right]}^{T}$$

The acceleration and velocity were calculated using EKF based on the simulation settings defined in Section 3.1. The errors resulting from these calculations are illustrated in Figure 2 and Figure 3. The mean and variance of these errors, when compared to the ground truth, are presented in Table 1. It is evident that the errors were erratically reduced compared to when the acceleration equations were used.

The initial conditions for the EKF were set as the state vector $x=\left[y_{1},0,0\right]$, covariance matrix $P=diag(\left[\begin{array}{ccc} \sigma_{y}^{2} & \sigma_{y}^{2} & \sigma_{y}^{2} \end{array}\right])$, measurement noise covariance $R=[\sigma_{y}]$, and process noise covariance *Q*, where $\sigma_{a}^{2}$ is the variance of the acceleration.

To derive the process noise covariance matrix *Q*, we started by considering that the process noise $w_{k}$ predominantly affected the acceleration component of the state vector. We modeled $w_{k}$ as follows:

(9) $$w_{k}=\left[\begin{array}{c} \frac{1}{2}\Delta t^{2}\\ \Delta t\\ 1 \end{array}\right]w_{a}$$

where $w_{a}$ represents Gaussian noise with variance $\sigma_{a}^{2}$. The process noise covariance matrix *Q* was computed as:

(10) $$Q=E\left[w_{k}w_{k}^{T}\right]=\sigma_{a}^{2}\left[\begin{array}{ccc} \frac{1}{4}\Delta t^{4} & \frac{1}{2}\Delta t^{3} & \frac{1}{2}\Delta t^{2}\\ \frac{1}{2}\Delta t^{3} & \Delta t^{2} & \Delta t\\ \frac{1}{2}\Delta t^{2} & \Delta t & 1 \end{array}\right]$$

We set $\sigma_{y}=0.01$ as the noise variance added to position points. We swept $\sigma_{a}^{2}$ from 1 to 200 and selected the value that minimized error values, which was found to be $\sigma_{a}^{2}=28$.

#### 3.1.2. Second-Order Regression (SOR)

To analyze the motion under constant acceleration, the SOR model can be employed to extract motion parameters. Given a set of *k* data points $\left\{\left(t_{1},y_{1}\right),\left(t_{2},y_{2}\right),\ldots ,\left(t_{k},y_{k}\right)\right\}$, where $t_{i}$ represents the time at which a measurement is taken, and $y_{i}$ represents the position of the object at time $t_{i}$, an SOR model can be employed to extract the motion parameters of an object under constant acceleration. The SOR model is formally represented by the equation:

(11) $$y=\beta_{0}+\beta_{1}t+\beta_{2}t^{2}$$

Here, *y* denotes the position of the object, *t* denotes time, $\beta_{0}$ is the intercept term corresponding to the initial position of the object ($y\left(0\right)$), $\beta_{1}$ is the coefficient of the linear term corresponding to the initial velocity of the object ($v\left(0\right)$), and $\beta_{2}$ is the coefficient of the quadratic term, representing half the acceleration $\left(\frac{1}{2}a\right)$.

To determine the values of $\beta_{0}$, $\beta_{1}$ and $\beta_{2}$ that best fit the observed data, we employed least squares. That approach minimized the sum of the squared differences between the observed positions ($y_{i}$) and the positions predicted by the model (${\hat{y}}_{i}$) over all *k* data points. The optimization problem can be formally stated as:

(12) $$\min_{\beta_{0},\beta_{1},\beta_{2}}\sum_{i=1}^{k}{\left(y_{i}-\left(\beta_{0}+\beta_{1}t_{i}+\beta_{2}t_{i}^{2}\right)\right)}^{2}$$

Solving this optimization problem allowed us to estimate ${\hat{\beta }}_{0}$, ${\hat{\beta }}_{1}$, and ${\hat{\beta }}_{2}$, from the initial position, initial velocity, and acceleration of the object, which could be directly inferred as follows: the initial position $y\left(0\right)$ was given by ${\hat{\beta }}_{0}$, the initial velocity by ${\hat{\beta }}_{1}$, and the acceleration *a* by $2{\hat{\beta }}_{2}$.

The acceleration and velocity were calculated using SOR, following the simulation settings defined in Section 3.1. The resulting errors from these calculations are depicted in Figure 2 and Figure 3. Additionally, the mean and variance of these errors, when compared to the ground truth, are presented in Table 1. Notably, the errors in this case were significantly reduced and exhibited greater accuracy compared to the previously mentioned method, bringing them much closer to the actual values.

Based on the experimental results provided, it is evident that the SOR outperforms other methods. Although the performance of the Extended Kalman Filter (EKF) and the SOR were comparable, the SOR method was selected for calculating acceleration due to the following reasons:

- The EKF requires a sufficient number of samples to initialize, converge, and provide accurate estimates. In datasets where some objects are only available in a limited number of frames, accurate acceleration estimation becomes challenging.
- The performance of the EKF is highly sensitive to the tuning of process and measurement noise covariance matrices [36]. Identifying optimal values can be time-consuming and necessitates domain expertise. Moreover, the optimal values may differ across various datasets because of variations in setup and sensors.
- The Extended Kalman Filter (EKF) assumes that the noise follows a Gaussian distribution [36], as its estimation framework is optimized for such conditions. In contrast, the Second-Order Regression (SOR) method is not limited by specific noise distribution assumptions. This flexibility makes SOR more robust and effective in scenarios where the noise is non-Gaussian or has an unknown distribution, which is common in real-world applications.

### 3.2. DAT

We present DAT, an end-to-end model appropriate for efficient and precise joint perception and forecasting within the realm of autonomous driving. Departing from the conventional approach of crafting separate models for individual subtasks, we embrace recent advancements in joint modeling through shared feature computation (end-to-end task). However, a primary drawback of such paradigms lies in their limitation when forecasting agents with both linear and nonlinear motion models, as noted by Peri et al. [4]. These limitations impede the efficacy of these approaches in motion forecasting tasks. In contrast, DAT tackles this challenge through a key enhancement: by incorporating the ability to learn the acceleration component of different agents, assuming a constant acceleration model between consecutive frames, it enhances the association between objects in future frames and their present counterparts, thereby enhancing trajectory forecasting accuracy. In the following, we first present DAT, which detects objects in present and N future frames, and then show how we built trajectories.

#### 3.2.1. Object Detection Module

We introduce significant innovations to the existing 3D object detector, CenterPoint [27]. The original detector employs a LiDAR-based backbone network, like VoxelNet or PointPillars [37,38], to generate a structured representation of the input point cloud. This representation is subsequently transformed into a top-down map view, where an image-based keypoint detector is utilized to locate object centers. For each detected center, the framework predicts supplementary object properties, including 3D size, orientation, and velocity.

Our detector further incorporates innovative acceleration (a) and initial velocity (v0) heads, allowing the network to learn essential parameters for modeling objects using kinematic motion equations. This enhancement enables CenterPoint to effectively manage both constant velocity and constant acceleration scenarios, greatly extending its ability to accurately model and predict real-world object movements with improved precision.

**Figure 4:**
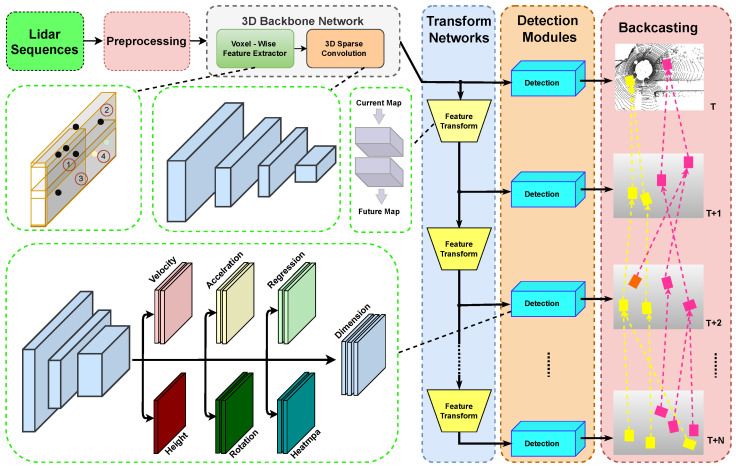
DAT: based on a LiDAR sequence, DAT detects objects in both the present frame (t) and future frames (up to t + T). These future detections are projected back to the current frame allowing for alignment with detections in the present moment.

The existing object detection model is tailored to detect objects within the present frame. While this approach efficiently captures an object’s location, it struggles to represent features that change over time. In our case, we want to detect cars and their future locations. These temporally distinct classes likely require different features for accurate detection. To address this challenge, our model incorporates a shallow network [39] that specifically transforms current object features into predicted future features (see Figure 4).

#### 3.2.2. Loss Function

Center heatmap loss: For each of the *T* object detection modules, let ${\hat{Y}}^{\left(t\right)}\in R^{W\times H\times K}$ represent the predicted heatmap for the *t*th module, and $Y\in R^{W\times H\times K}$ be the ground truth heatmap. We employ the focal loss [40,41] for heatmap supervision. The total heatmap loss across all *T* modules is defined as:

(13) $$L_{\mathrm{heatmap}}=\sum_{t=1}^{T}\mathrm{FocalLoss}\left({\hat{Y}}^{\left(t\right)},Y\right)$$

Shared regression loss: The regression heads are shared across all *T* detection modules. The shared regression loss is used to supervise sub-voxel location refinement, object size, height-above-ground, rotation, acceleration, and initial velocity predictions. All outputs are trained using an L1 loss centered at the ground-truth location. To more effectively manage objects of diverse shapes, size regression is performed using a logarithmic scale. During the inference phase, object properties are retrieved by mapping them to the dense regression head outputs at the peak position of each object. The regression loss is defined as follows:

(14) $$L_{\mathrm{regression}}=\frac{1}{N}\sum_{i=1}^{N}\left|{\hat{r}}_{i}-r_{i}\right|$$

where ${\hat{r}}_{i}$ represents the predicted values for the various regression targets, and $r_{i}$ represents the corresponding ground-truth values.

Overall loss function: Since we assign equal weights for the heatmap and regression losses, the total loss function is formulated as follows:

(15) $$L_{\mathrm{total}}=L_{\mathrm{heatmap}}+L_{\mathrm{regression}}$$

### 3.3. From Detection to Trajectory Forecasting

DAT addresses the challenge of object forecasting by estimating the future positions of objects based on their observations at the initial time step ($t_{obs}$). We utilized adapted LiDAR detectors to estimate object positions over *T* future, unobserved LiDAR scans, using ground-truth data for training purposes. To estimate future object trajectories, our network was trained to additionally predict acceleration vectors for each detection in every future frame.

For linking objects across different frames, FaF [31] proposed an architecture that directly forecasted velocity into the future based on current-frame detections. FutureDet [4], however, adopts an inverse approach. It simultaneously detects objects at both present and subsequent timestamps and estimates velocity to associate them with their corresponding positions in the past. This essentially links objects from the future to the present frame, assuming constant velocity between frames. DAT follows a similar linking strategy (future to present) but incorporates a constant acceleration motion model for improved accuracy.

Trajectory construction involves aligning all trajectories with the object detected in the present LiDAR scan. To achieve this, each detected object in the subsequent frame (n) is connected with the preceding frame (m) by employing the constant acceleration model. Subsequently, the spatial distance between the detected object at time n and all other detected objects is computed, and the nearest object is then selected.

## 4. Experimental Results

We validated the effectiveness of our method using the NuScenes dataset [8], which is a comprehensive, real-world driving dataset.

### 4.1. Dataset

Our experimental evaluation was conducted using the NuScenes dataset [8], which consists of 1000 log segments, each with a duration of 20 s. Each snippet is equipped with 32-beam LiDAR sweeps operating at a frequency of 20 Hz, along with corresponding 3D object annotations. We used the Trainval split, which includes 700 scenes for training and 150 scenes for validation. We adhered to the official protocol and assessed forecasting for the car class, predicting up to 3 s into the future.

### 4.2. Implementation Details

DAT was trained to detect objects in both current and future frames by encoding the aggregated point cloud sequences using the VoxelNet and PointPillars backbones [37,38]. We employed ground-truth sampling (also known as copy–paste) augmentation [42] to enhance the diversity of training trajectories, leading to significant improvements in both linear and nonlinear forecasting performance. All models were trained using the PyTorch toolbox for 20 epochs with the Adam optimizer, utilizing a learning rate of $1\times 10^{-4}$ and a weight decay of 0.01. The models were trained on two A4000 GPUs.

### 4.3. Evaluation Metrics

We adopted the detection and forecasting metrics proposed by Peri et al. [4] to ensure a fair comparison with state-of-the-art methods. Mean Average Precision (AP) [43] was utilized for object detection, while Forecasting Mean Average Precision (${mAP}_{f}$) was employed for trajectory prediction. AP is defined as the area under the IoU-based Precision–Recall curve. Note that AP measures the performance of the object detection task alone. ${mAP}_{f}$ is a metric used to evaluate the accuracy of forecasting in the context of joint detection and forecasting tasks. It considers both the detection of objects in the current frame and the forecasting of their future positions. We further categorized the dataset into three subclasses: static cars, cars with linear motion, and cars with nonlinear motion [4]. For these categories, we report ${AP}_{f}$ and ${AP}_{det}$. Additionally, we computed the mean Average Precision for forecasting (${mAP}_{f}$) as ${mAP}_{f}=\frac{1}{3}\times \left({AP}_{f}^{lin.}+{AP}_{f}^{non-lin.}+{AP}_{f}^{stat.}\right)$. Similarly, the mean Average Precision for detection (${mAP}_{det}$) was calculated as the average ${AP}_{det}$ across the three subclasses [4].

### 4.4. Comparison with State of the Art

In this section, we provide a comparison between our proposed forecasting approach and cutting-edge methods, using two evaluation metrics, $AP_{f}$ and $AP_{det}$, and classes were further divided into three sub-classes: static, linear, and nonlinear [4]. These comparisons were made under the evaluation settings of top-K forecasting, specifically for K=1 and K=5. Our network was trained using two feature extraction techniques, VoxelNet and PointPillars. Additionally, we developed a variant of our model that incorporated road masks as an extra input layer into the Bird’s Eye View (BEV) feature representation, following the sparse voxel backbone processing. As illustrated in Table 2, DAT surpassed all previous state-of-the-art methods in terms of both forecasting and detection capabilities. Specifically, our method achieved a 4% improvement in $mAP_{det}$ and a significant increase in $mAP_{f}$ of 13% for K=1 and 27% for K=5.

For linear moving objects under the K=1 setting, our method achieved a remarkable $AP_{f}$ of 30.1%, which was a significant improvement over the best previous result reported by TrajectoryNAS [35] at 26%, marking an improvement of approximately 15.8%. Similarly, under the K=5 setting, our method extended its lead with an $AP_{f}$ of 36.3%, compared to the highest $AP_{f}$ of 29.2% from TrajectoryNAS, translating into a substantial improvement of 24.3%.

When focusing on nonlinear moving objects, our approach demonstrated even more pronounced advancements. For K=1, our method recorded an $AP_{f}$ of 18.7%, surpassing the nearest competitor, TrajectoryNAS [35], which achieved a $AP_{f}$ of 10.3%, equating to an 82.0% improvement. For K=5, our method showcased an $AP_{f}$ of 24%, significantly outperforming TrajectoryNAS, which had an earlier best of 12.1%.

DAT performed well because it was specifically designed to learn acceleration. This enabled it to model object movements more accurately (in backcasting). Integrating road masks as an additional input channel into our model did not significantly affect the results. This can be attributed to two main factors: first, the map information does not offer substantial new insights, as it can be effectively inferred from the raw LiDAR data; second, certain map data suffer from significant alignment inaccuracies with the LiDAR data [1,2].

### 4.5. Ablation Study

To investigate the influence of windowing length on our model’s performance, utilized for acceleration calculation in the ground truth, we conducted an ablation study, the results of which are presented in Table 3. Our results indicate that while detection accuracy remained consistent across different window lengths, forecasting accuracy varied. Notably, a window length of five resulted in inferior forecasting compared to other lengths.

The choice of window length significantly impacted the model’s ability to capture motion dynamics accurately. A suitable window length balanced noise reduction and preserved essential motion patterns. Our analysis revealed that the model’s overall accuracy was relatively unaffected by changes in window length, suggesting its robustness in capturing diverse motion characteristics.

The insensitivity of our model to variations in window length underscores its potential for generalization across different datasets and sensor configurations. This is crucial as different datasets have varying update rates, requiring adjustments in window length for consistent acceleration calculations. Our results demonstrate the model’s adaptability and reliability in handling diverse data conditions.

### 4.6. Qualitative Results

**Figure 5:**
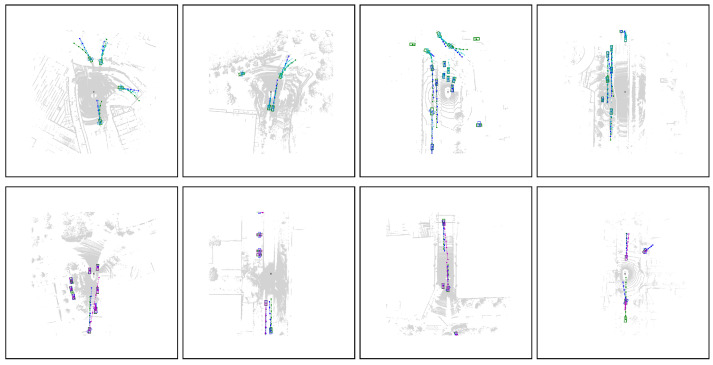
Qualitative evaluation of trajectory forecasts using DAT. In the first row, ground-truth trajectories are depicted in green, the highest confidence forecast in blue, and other potential future trajectories in cyan. The second row compares the highest confidence forecasts of DAT (blue) with those of TrajectoryNAS (magenta), alongside the ground-truth trajectories (green). The results illustrate that DAT predictions are closer to the ground truth.

The qualitative results of our proposed model are presented in the Figure 5. These results demonstrate that by integrating acceleration representations, DAT effectively depicts a wide range of potential future scenarios. The model successfully captured the motion of static, linear, and nonlinear objects. In the first row, various scenes are depicted, including ground-truth trajectories, the forecasted trajectory with the highest confidence, and additional predicted trajectories. The second row compares our DAT model and TrajectoryNAS [35], highlighting that our model’s predicted trajectories closely align with the ground truth.

## 5. Conclusions

In this paper, we introduced the Deep learning-based Acceleration-aware Trajectory forecasting (DAT) model, a novel end-to-end framework for trajectory forecasting that directly utilizes LiDAR sensor data. Our primary contribution is the development of a forecasting method that incorporates the acceleration of surrounding agents, a key factor in improving the accuracy of trajectory predictions, particularly for objects exhibiting nonlinear motion. Unlike existing methods, which struggle with agents displaying complex dynamics, DAT’s integration of acceleration significantly enhances its predictive capability, especially in dynamic and unpredictable traffic environments.

The practical advantages of DAT extend to real-world autonomous driving applications. Its ability to model and predict complex, nonlinear motion patterns allows for faster and more accurate responses to sudden changes in the environment, such as abrupt lane changes or unexpected stops. This can significantly reduce the risk of collisions and improve the overall safety of autonomous vehicles. Furthermore, DAT’s robust approach to estimating ground-truth acceleration using the Second-Order Regression (SOR) method ensures the reliability and adaptability of the model in diverse driving conditions.

By conducting comprehensive experiments on the NuScenes dataset, we demonstrated that DAT outperformed state-of-the-art methods, particularly in handling diverse motion patterns. This positions DAT as a promising solution for real-world deployment in autonomous driving systems, where both accuracy and the ability to react to complex, real-time traffic scenarios are critical. By improving trajectory forecasting, DAT has the potential to enhance decision-making, contributing to safer and more efficient autonomous driving in dynamic environments.

## Figures and Tables

**Table 1:** Comparison of velocity and acceleration error. The cyan color specified the best errors gathered.

	$V_{0}$		*A*	
	$\mu_{v}$	$\sigma_{v}^{2}$	$\mu_{a}$	$\sigma_{a}^{2}$
SOR	0.02	$0.03^{2}$	0.04	$0.06^{2}$
EKF	0.12	$0.17^{2}$	0.30	$0.79^{2}$
Equation	0.30	$0.19^{2}$	1.57	$1.84^{2}$

**Table 2:** Evaluation of the car object detection and trajectory forecasting pipeline on the NuScenes datasets. Rows with cyan color are our model’s results.

	K=1								K=5							
	${\mathrm{AP}}^{\mathrm{stat}.}$		${\mathrm{AP}}^{\mathrm{lin}.}$		${\mathrm{AP}}^{\mathrm{non}-\mathrm{lin}.}$		mAP		${\mathrm{AP}}^{\mathrm{stat}.}$		${\mathrm{AP}}^{\mathrm{lin}.}$		${\mathrm{AP}}^{\mathrm{non}-\mathrm{lin}.}$		mAP	
	${\mathrm{AP}}_{\det.}$	${\mathrm{AP}}_{f}$	${\mathrm{AP}}_{\det.}$	${\mathrm{AP}}_{f}$	${\mathrm{AP}}_{\det.}$	${\mathrm{AP}}_{f}$	${\mathrm{AP}}_{\det.}$	${\mathrm{AP}}_{f}$	${\mathrm{AP}}_{\det.}$	${\mathrm{AP}}_{f}$	${\mathrm{AP}}_{\det.}$	${\mathrm{AP}}_{f}$	${\mathrm{AP}}_{\det.}$	${\mathrm{AP}}_{f}$	${\mathrm{AP}}_{\det.}$	${\mathrm{AP}}_{f}$
Detection + Constant Velocity	70.3	66.0	65.8	21.2	90.0	6.5	75.4	31.12	70.3	66.0	65.8	21.2	90.0	6.5	75.4	31.2
Detection + Forecast (Luo et al. [31])	69.1	64.7	66.1	22.2	86.3	7.5	73.8	31.5	69.1	64.7	66.1	22.2	86.3	7.5	73.8	31.5
FutureDet (Peri et al. [4])	70.0	65.5	62.9	24.9	**91.8**	10.1	74.9	33.5	70.1	67.3	62.9	27.7	**91.7**	11.7	74.9	35.6
FutureDet–PointPillars (Peri et al. [4])	70.1	64.1	63.4	24.8	92.4	9.6	75.4	32.8	70.7	67.5	63.4	28.8	92.0	11.9	75.4	36.1
FutureDet + Map (Peri et al. [4])	70.2	65.5	62.7	24.3	91.7	9.4	74.9	33.1	70.2	67.5	62.7	27.1	91.7	11.0	74.9	35.2
TrajectoryNAS (Sharifi et al. [35])	71.2	65.6	63.8	26	91.2	10.3	75	34	71	67.4	63.8	29.2	91.1	12.1	75.3	36.2
Ours	72.1	**66.2**	**70.8**	**30.1**	91.3	**18.7**	**78.0**	**38.3**	72.1	**69.4**	**70.8**	**36.3**	91.3	23.5	**78.0**	**43.0**
Ours + PointPillars	70.0	62.7	65.5	27.1	89.3	17.5	75.0	35.8	70.0	66.5	65.5	33.9	89.3	**24.0**	75.0	41.1
Ours + MAP	**72.3**	66.0	70.2	29.6	91.5	18.1	**78.0**	37.9	**72.3**	69.3	70.2	35.5	91.5	22.8	**78.0**	42.5

**Table 3:** Ablation study on the impact of varying the windowing length, employed in calculating acceleration within the ground truth, on the accuracy of both forecasting and detection.

Window Length	K=1								K=5							
	${\mathrm{AP}}^{\mathrm{stat}.}$		${\mathrm{AP}}^{\mathrm{lin}.}$		${\mathrm{AP}}^{\mathrm{non}-\mathrm{lin}.}$		mAP		${\mathrm{AP}}^{\mathrm{stat}.}$		${\mathrm{AP}}^{\mathrm{lin}.}$		${\mathrm{AP}}^{\mathrm{non}-\mathrm{lin}.}$		mAP	
	${\mathrm{AP}}_{\det.}$	${\mathrm{AP}}_{f}$	${\mathrm{AP}}_{\det.}$	${\mathrm{AP}}_{f}$	${\mathrm{AP}}_{\det.}$	${\mathrm{AP}}_{f}$	${\mathrm{AP}}_{\det.}$	${\mathrm{AP}}_{f}$	${\mathrm{AP}}_{\det.}$	${\mathrm{AP}}_{f}$	${\mathrm{AP}}_{\det.}$	${\mathrm{AP}}_{f}$	${\mathrm{AP}}_{\det.}$	${\mathrm{AP}}_{f}$	${\mathrm{AP}}_{\det.}$	${\mathrm{AP}}_{f}$
5	72.0	65.7	69.8	29.5	91.0	18.5	77.6	37.9	72.0	69.2	69.8	35.0	91.0	23.0	77.6	42.4
10	72.0	65.8	71.1	29.9	91.1	18.8	78.0	38.1	72.0	69.2	71.1	35.7	91.1	23.2	78.0	42.7
15	72.1	66.2	70.8	30.1	91.3	18.7	78.0	38.3	72.1	69.4	70.8	36.3	91.3	23.5	78.0	43.0

## Data Availability

Data available on request due to restrictions (e.g., privacy, legal or ethical reasons).

## Appendix A. Velocity and Acceleration Variance

### Appendix A.1. Acceleration Variance

**Proof.** Given the equation for acceleration *a*:

(A1) $$a=\frac{2(y_{2}-y_{0})}{\left(t_{2}-t_{1}\right)\left(t_{2}-t_{0}\right)}-\frac{2(y_{1}-y_{0})}{\left(t_{1}-t_{0}\right)\left(t_{2}-t_{1}\right)}$$

where $y_{0}$, $y_{1}$, and $y_{2}$ are noisy observations, perturbed by Gaussian noise with mean zero and variance $\sigma^{2}$, let the noisy *y* values be expressed as $y_{i}=y_{i}^{\mathrm{true}}+\epsilon_{i}$, where $\epsilon_{i}∼N\left(0,\sigma^{2}\right)$. The goal is to derive the variance of *a* under these noise conditions. Rewriting *a* in terms of the noisy *y* values, we obtain:

(A2) $$a=\frac{2(\left(y_{2}^{\mathrm{true}}+\epsilon_{2}\right)-\left(y_{0}^{\mathrm{true}}+\epsilon_{0}\right))}{\left(t_{2}-t_{1}\right)\left(t_{2}-t_{0}\right)}-\frac{2(\left(y_{1}^{\mathrm{true}}+\epsilon_{1}\right)-\left(y_{0}^{\mathrm{true}}+\epsilon_{0}\right))}{\left(t_{1}-t_{0}\right)\left(t_{2}-t_{1}\right)}$$

which simplifies to:

(A3) $$a=a^{\mathrm{true}}+\left(\frac{2(\epsilon_{2}-\epsilon_{0})}{\left(t_{2}-t_{1}\right)\left(t_{2}-t_{0}\right)}-\frac{2(\epsilon_{1}-\epsilon_{0})}{\left(t_{1}-t_{0}\right)\left(t_{2}-t_{1}\right)}\right)$$

Since $a^{\mathrm{true}}$ is constant, the variance of *a* is determined by the noise terms. Thus, the variance of *a* is:

(A4) $$\mathrm{Var}\left(a\right)=\mathrm{Var}\left(\frac{2(\epsilon_{2}-\epsilon_{0})}{\left(t_{2}-t_{1}\right)\left(t_{2}-t_{0}\right)}-\frac{2(\epsilon_{1}-\epsilon_{0})}{\left(t_{1}-t_{0}\right)\left(t_{2}-t_{1}\right)}\right)$$

Using the property that $\epsilon_{0}$, $\epsilon_{1}$, and $\epsilon_{2}$ are independent Gaussian variables with variance $\sigma^{2}$, we obtain:

(A5) $$\mathrm{Var}\left(a\right)=\frac{8\sigma^{2}}{{\left(\left(t_{2}-t_{1}\right)\left(t_{2}-t_{0}\right)\right)}^{2}}+\frac{8\sigma^{2}}{{\left(\left(t_{1}-t_{0}\right)\left(t_{2}-t_{1}\right)\right)}^{2}}$$

Thus, the variance of *a* is:

(A6) $$\mathrm{Var}\left(a\right)=\frac{8\sigma^{2}}{{\left(t_{2}-t_{1}\right)}^{2}{\left(t_{2}-t_{0}\right)}^{2}}+\frac{8\sigma^{2}}{{\left(t_{1}-t_{0}\right)}^{2}{\left(t_{2}-t_{1}\right)}^{2}}$$

□

### Appendix A.2. Velocity Variance

**Proof.** Given the equation for velocity $v_{0}$:

(A7) $$v_{0}=\frac{2\left(y_{1}-y_{0}\right)\left(t_{2}-t_{0}\right)}{\left(t_{2}-t_{1}\right)\left(t_{1}-t_{0}\right)}-\frac{2\left(y_{2}-y_{0}\right)\left(t_{1}-t_{0}\right)}{\left(t_{2}-t_{1}\right)\left(t_{2}-t_{0}\right)}$$

where, again, $y_{0}$, $y_{1}$, and $y_{2}$ are noisy with Gaussian noise, we express the noisy *y* values as $y_{i}=y_{i}^{\mathrm{true}}+\epsilon_{i}$, where $\epsilon_{i}∼N\left(0,\sigma^{2}\right)$, and proceed to derive the variance of $v_{0}$. Rewriting $v_{0}$ in terms of the noisy *y* values yields:

(A8) $$v_{0}=\frac{2\left(\left(y_{1}^{\mathrm{true}}+\epsilon_{1}\right)-\left(y_{0}^{\mathrm{true}}+\epsilon_{0}\right)\right)\left(t_{2}-t_{0}\right)}{\left(t_{2}-t_{1}\right)\left(t_{1}-t_{0}\right)}-\frac{2\left(\left(y_{2}^{\mathrm{true}}+\epsilon_{2}\right)-\left(y_{0}^{\mathrm{true}}+\epsilon_{0}\right)\right)\left(t_{1}-t_{0}\right)}{\left(t_{2}-t_{1}\right)\left(t_{2}-t_{0}\right)}$$

Simplifying, we obtain:

(A9) $$v_{0}=v_{0}^{\mathrm{true}}+\left(\frac{2\left(\epsilon_{1}-\epsilon_{0}\right)\left(t_{2}-t_{0}\right)}{\left(t_{2}-t_{1}\right)\left(t_{1}-t_{0}\right)}-\frac{2\left(\epsilon_{2}-\epsilon_{0}\right)\left(t_{1}-t_{0}\right)}{\left(t_{2}-t_{1}\right)\left(t_{2}-t_{0}\right)}\right)$$

Thus, the variance of $v_{0}$ is:

(A10) $$\mathrm{Var}\left(v_{0}\right)=\mathrm{Var}\left(\frac{2\left(\epsilon_{1}-\epsilon_{0}\right)\left(t_{2}-t_{0}\right)}{\left(t_{2}-t_{1}\right)\left(t_{1}-t_{0}\right)}-\frac{2\left(\epsilon_{2}-\epsilon_{0}\right)\left(t_{1}-t_{0}\right)}{\left(t_{2}-t_{1}\right)\left(t_{2}-t_{0}\right)}\right)$$

Using the same variance properties as before, we have:

(A11) $$\mathrm{Var}\left(v_{0}\right)=\frac{8\sigma^{2}{\left(t_{2}-t_{0}\right)}^{2}}{{\left(\left(t_{2}-t_{1}\right)\left(t_{1}-t_{0}\right)\right)}^{2}}+\frac{8\sigma^{2}{\left(t_{1}-t_{0}\right)}^{2}}{{\left(\left(t_{2}-t_{1}\right)\left(t_{2}-t_{0}\right)\right)}^{2}}$$

Thus, the variance of $v_{0}$ is:

(A12) $$\mathrm{Var}\left(v_{0}\right)=\frac{8\sigma^{2}{\left(t_{2}-t_{0}\right)}^{2}}{{\left(t_{2}-t_{1}\right)}^{2}{\left(t_{1}-t_{0}\right)}^{2}}+\frac{8\sigma^{2}{\left(t_{1}-t_{0}\right)}^{2}}{{\left(t_{2}-t_{1}\right)}^{2}{\left(t_{2}-t_{0}\right)}^{2}}$$

□

## References

[1] Li L.L., Yang B., Liang M., Zeng W., Ren M., Segal S., Urtasun R. (2020). End-to-end contextual perception and prediction with interaction transformer. Proceedings of the 2020 IEEE/RSJ International Conference on Intelligent Robots and Systems (IROS).

[2] Liang M., Yang B., Zeng W., Chen Y., Hu R., Casas S., Urtasun R. Pnpnet: End-to-end perception and prediction with tracking in the loop. Proceedings of the IEEE/CVF Conference on Computer Vision and Pattern Recognition.

[3] Marchetti F., Becattini F., Seidenari L., Del Bimbo A. (2020). Multiple trajectory prediction of moving agents with memory augmented networks. IEEE Trans. Pattern Anal. Mach. Intell..

[4] Peri N., Luiten J., Li M., Ošep A., Leal-Taixé L., Ramanan D. Forecasting from lidar via future object detection. Proceedings of the IEEE/CVF Conference on Computer Vision and Pattern Recognition.

[5] Moshayedi A.J., Roy A.S., Kolahdooz A., Shuxin Y. (2022). Deep learning application pros and cons over algorithm deep learning application pros and cons over algorithm. EAI Endorsed Trans. AI Robot..

[6] Geiger A., Lenz P., Urtasun R. (2012). Are we ready for autonomous driving? the kitti vision benchmark suite. Proceedings of the 2012 IEEE Conference on Computer Vision and Pattern Recognition.

[7] Chang M.F., Lambert J., Sangkloy P., Singh J., Bak S., Hartnett A., Wang D., Carr P., Lucey S., Ramanan D. Argoverse: 3d tracking and forecasting with rich maps. Proceedings of the IEEE/CVF Conference on Computer Vision and Pattern Recognition.

[8] Caesar H., Bankiti V., Lang A.H., Vora S., Liong V.E., Xu Q., Krishnan A., Pan Y., Baldan G., Beijbom O. nuscenes: A multimodal dataset for autonomous driving. Proceedings of the IEEE/CVF Conference on Computer Vision and Pattern Recognition.

[9] Draper N.R., Smith H. (1998). Applied Regression Analysis.

[10] Xu Y., Lin J., Shi J., Zhang G., Wang X., Li H. (2022). Robust self-supervised lidar odometry via representative structure discovery and 3d inherent error modeling. IEEE Robot. Autom. Lett..

[11] Gu C., Shokry A., Youssef M. (2021). The effect of ground truth accuracy on the evaluation of localization systems. Proceedings of the IEEE INFOCOM 2021-IEEE Conference on Computer Communications.

[12] Leon F., Gavrilescu M. (2021). A review of tracking and trajectory prediction methods for autonomous driving. Mathematics.

[13] Moshayedi A.J., Roy A.S., Liao L., Khan A.S., Kolahdooz A., Eftekhari A. (2024). Design and Development of FOODIEBOT Robot: From Simulation to Design. IEEE Access.

[14] Chai Y., Sapp B., Bansal M., Anguelov D. MultiPath: Multiple Probabilistic Anchor Trajectory Hypotheses for Behavior Prediction. Proceedings of the Conference on Robot Learning, PMLR.

[15] Cui H., Radosavljevic V., Chou F.C., Lin T.H., Nguyen T., Huang T.K., Schneider J., Djuric N. (2019). Multimodal trajectory predictions for autonomous driving using deep convolutional networks. Proceedings of the 2019 International Conference on Robotics and Automation (ICRA).

[16] Phan-Minh T., Grigore E.C., Boulton F.A., Beijbom O., Wolff E.M. Covernet: Multimodal behavior prediction using trajectory sets. Proceedings of the IEEE/CVF Conference on Computer Vision and Pattern Recognition.

[17] Gao J., Sun C., Zhao H., Shen Y., Anguelov D., Li C., Schmid C. Vectornet: Encoding hd maps and agent dynamics from vectorized representation. Proceedings of the IEEE/CVF Conference on Computer Vision and Pattern Recognition.

[18] Liang M., Yang B., Hu R., Chen Y., Liao R., Feng S., Urtasun R. (2020). Learning lane graph representations for motion forecasting. Proceedings of the Computer Vision—ECCV 2020: 16th European Conference, Proceedings.

[19] Ye M., Cao T., Chen Q. Tpcn: Temporal point cloud networks for motion forecasting. Proceedings of the IEEE/CVF Conference on Computer Vision and Pattern Recognition.

[20] Han K., Wang Y., Chen H., Chen X., Guo J., Liu Z., Tang Y., Xiao A., Xu C., Xu Y. (2022). A survey on vision transformer. IEEE Trans. Pattern Anal. Mach. Intell..

[21] Yuan Y., Weng X., Ou Y., Kitani K.M. Agentformer: Agent-aware transformers for socio-temporal multi-agent forecasting. Proceedings of the IEEE/CVF International Conference on Computer Vision.

[22] Khandelwal S., Qi W., Singh J., Hartnett A., Ramanan D. (2020). What-if motion prediction for autonomous driving. arXiv.

[23] Weng X., Ivanovic B., Kitani K., Pavone M. Whose track is it anyway? Improving robustness to tracking errors with affinity-based trajectory prediction. Proceedings of the IEEE/CVF Conference on Computer Vision and Pattern Recognition.

[24] Wang W., Chang X., Yang J., Xu G. (2022). LiDAR-based dense pedestrian detection and tracking. Appl. Sci..

[25] Wang S., Sun Y., Liu C., Liu M. (2020). Pointtracknet: An end-to-end network for 3-d object detection and tracking from point clouds. IEEE Robot. Autom. Lett..

[26] Guo X., Gu J., Guo S., Xu Z., Yang C., Liu S., Cheng L., Huang K. (2020). 3D object detection and tracking based on streaming data. Proceedings of the 2020 IEEE International Conference on Robotics and Automation (ICRA).

[27] Yin T., Zhou X., Krahenbuhl P. Center-based 3d object detection and tracking. Proceedings of the IEEE/CVF Conference on Computer Vision and Pattern Recognition.

[28] Li X., Guivant J.E. (2023). Efficient and Accurate Object Detection With Simultaneous Classification and Tracking Under Limited Computing Power. IEEE Trans. Intell. Transp. Syst..

[29] Simon M., Amende K., Kraus A., Honer J., Samann T., Kaulbersch H., Milz S., Michael Gross H. Complexer-yolo: Real-time 3d object detection and tracking on semantic point clouds. Proceedings of the IEEE/CVF Conference on Computer Vision and Pattern Recognition Workshops.

[30] Weng X., Yuan Y., Kitani K. (2021). PTP: Parallelized tracking and prediction with graph neural networks and diversity sampling. IEEE Robot. Autom. Lett..

[31] Luo W., Yang B., Urtasun R. Fast and furious: Real time end-to-end 3d detection, tracking and motion forecasting with a single convolutional net. Proceedings of the IEEE conference on Computer Vision and Pattern Recognition.

[32] Casas S., Luo W., Urtasun R. Intentnet: Learning to predict intention from raw sensor data. Proceedings of the Conference on Robot Learning, PMLR.

[33] Zeng W., Luo W., Suo S., Sadat A., Yang B., Casas S., Urtasun R. End-to-end interpretable neural motion planner. Proceedings of the IEEE/CVF Conference on Computer Vision and Pattern Recognition.

[34] Weng X., Wang J., Levine S., Kitani K., Rhinehart N. Inverting the pose forecasting pipeline with SPF2: Sequential pointcloud forecasting for sequential pose forecasting. Proceedings of the Conference on Robot Learning, PMLR.

[35] Sharifi A.A., Zoljodi A., Daneshtalab M. (2024). TrajectoryNAS: A Neural Architecture Search for Trajectory Prediction. Sensors.

[36] Simon D. (2006). Optimal State Estimation: Kalman, H Infinity, and Nonlinear Approaches.

[37] Lang A.H., Vora S., Caesar H., Zhou L., Yang J., Beijbom O. Pointpillars: Fast encoders for object detection from point clouds. Proceedings of the IEEE/CVF Conference on Computer Vision and Pattern Recognition.

[38] Zhou Y., Tuzel O. Voxelnet: End-to-end learning for point cloud based 3d object detection. Proceedings of the IEEE Conference on Computer Vision and Pattern Recognition.

[39] Luc P., Couprie C., Lecun Y., Verbeek J. Predicting future instance segmentation by forecasting convolutional features. Proceedings of the European Conference on Computer Vision (ECCV).

[40] Zhou X., Wang D., Krähenbühl P. (2019). Objects as points. arXiv.

[41] Law H., Deng J. Cornernet: Detecting objects as paired keypoints. Proceedings of the European Conference on Computer Vision (ECCV).

[42] Zhu B., Jiang Z., Zhou X., Li Z., Yu G. (2019). Class-balanced grouping and sampling for point cloud 3d object detection. arXiv.

[43] Everingham M., Van Gool L., Williams C.K., Winn J., Zisserman A. (2010). The pascal visual object classes (voc) challenge. Int. J. Comput. Vis..

